# Auricular therapy for polycystic ovary syndrome: A protocol for a systematic review and meta-analysis

**DOI:** 10.1097/MD.0000000000023396

**Published:** 2020-12-04

**Authors:** Yangyang Li, Xiaoying Zheng, Yingji Wang, Yan Li

**Affiliations:** a Heilongjiang University of Chinese Medicine.; b First Affiliated Hospital, Heilongjiang University of Chinese Medicine.; c College of Pharmacy, Harbin Medical University, Harbin, China.

**Keywords:** auricular therapy, meta-analysis, polycystic ovary syndrome, systematic review

## Abstract

**Background:**

This systematic review protocol aims to describe a meta-analysis to assess the effectiveness and safety of auricular therapy for patients with polycystic ovary syndrome (PCOS).

**Methods:**

Randomized controlled trials of auricular therapy in treating PCOS will be searched in PubMed, Embase, Web of Science, China National Knowledge Infrastructure, Wan-Fang Database, and Chinese Scientific Journal Database. The primary outcome is the body mass index. The study selection, data extraction, and study quality evaluation will be performed independently by 2 researchers. A meta-analysis will be performed using RevMan V5.3 statistical software if possible; otherwise, descriptive analysis or subgroup analysis will be conducted. The quality of evidence for outcomes will be assessed with the Grading of Recommendations Assessment, Development and Evaluation approach.

**Results:**

This study will evaluate the effect and safety of auricular therapy in treating PCOS.

**Conclusions:**

The evidence we generated from the present study will provide more options for PCOS management in clinical practice.

**The registration DOI:**

10.17605/OSF.IO/VBPSM.

## 1 Introduction

Polycystic ovary syndrome (PCOS) is a common endocrine disorder impacting 5% to 20% of reproductive women, its main symptoms including infertility, menstrual dysfunction, hirsutism, and obesity, which not only cause physical dysfunction but also psychosocial stress.^[1]^ The endocrine society suggested lifestyle therapy (calorie-restricted diet and exercise) with the objective of weight loss should also be the first-line treatment in the presence of overweight/obesity since weight loss is likely beneficial for both reproductive and metabolic dysfunction in this setting.^[2]^

Auricular therapy (AT) is based both upon the theory of Traditional Chinese Medicine and upon neurological reflex therapies that were discovered in Europe. It has been shown favorable effects in depression,^[3,4]^ pain relief,^[5]^ alleviation of addictions,^[6]^ and body weight reduction.^[7]^

There are clinical trials regarding AT in treating PCOS,^[8–11]^ however, the effect of AT on PCOS is still uncertain. To the best of our knowledge, there have been no systematic reviews that have been designed specifically to investigate the efficacy of AT for PCOS. Therefore, it is of great importance to evaluate the effectiveness and safety of AT in the treatment of PCOS through meta-analysis and provide a sufficient basis for its clinical application.

## 2 Methods and analysis

### 2.1 Study registration

This systematic review protocol has been registered on OSF. The registration DOI was 10.17605/OSF.IO/VBPSM. The protocol followed the Preferred Reporting Items for Systematic Reviews and Meta-Analysis Protocol (PRISMA-P) statement guidelines.^[12]^

### 2.2 Eligibility criteria

#### 2.2.1 Types of studies

The present systematic review will include randomized controlled trials (RCTs) only.

#### 2.2.2 Types of participants

The review will include patients diagnosed with specified diagnosis criteria of PCOS, e.g., revised Rotterdam Criteria 2003, National Institute of Health (NIH) 1990 criteria, or Androgen Excess and Polycystic Ovary Syndrome (AE-PCOS) Society criteria.^[13–15]^

#### 2.2.3 Type of interventions

In the present study, the intervention used in the treatment group should be AT or combined with medication or other treatments. AT includes auricular acupuncture, auricular acupressure, and treatment stimulating auricular acupoints according to Chinese Medicine nomenclature. The control group could be one or more of the following treatments: sham AT, placebo, medication, lifestyle modification, other complementary and alternative medicine treatment, or no treatment.

#### 2.2.4 Types of outcome measures

The primary outcome is the body mass index (BMI). The secondary outcomes include:

1. Hormonal profile, e.g., testosterone (T), follicle-stimulating hormone (FSH) and luteinizing hormone (LH);
2. Metabolic Characteristics including fasting insulin, fasting glucose, insulin resistance;
3. Clinical reproductive outcomes including pregnancy rate, ovulation rate, and menstrual frequency;
4. Adverse events.

### 2.3 Search strategy

The reviewers will conduct a systematic literature search in the following electronic databases: PubMed, Embase, Web of Science, China National Knowledge Infrastructure (CNKI), Wan-Fang Database, and Chinese Scientific Journal Database (VIP database). The search terms include auricular acupuncture, auricular acupressure, ear acupuncture, auricular acupoints, polycystic ovary syndrome, and randomized controlled trial. The search dates will be set from the inception to September 2020.

### 2.4 Data selection and extraction

#### 2.4.1 Study selection

**Figure 1:**
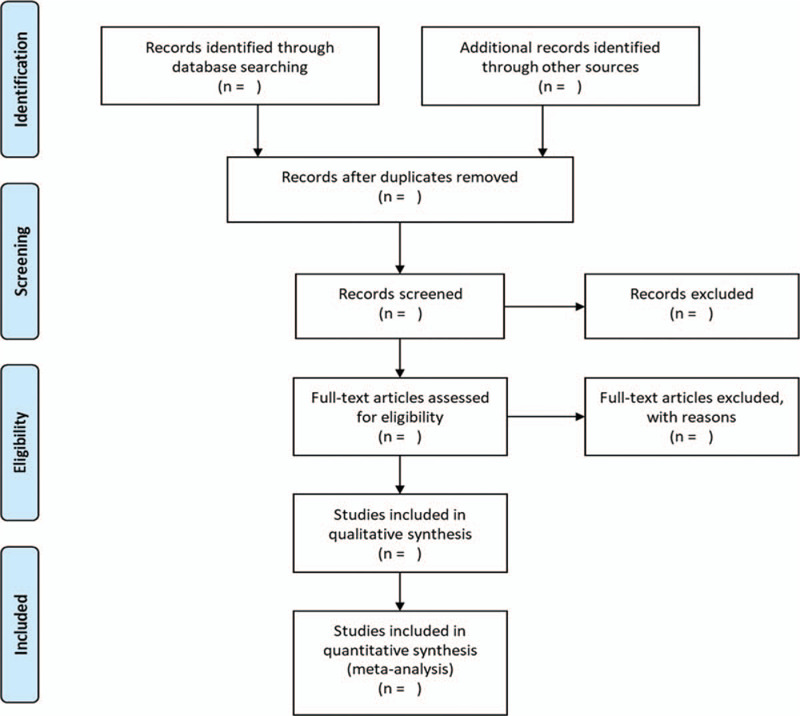
Flow chart of study selection.

Two investigators (YYL and XYZ) will independently screen all titles and abstracts to get qualified studies, and then excluded duplications. After that, the full text of all potential studies will be checked for further screening. We will record all removed studies with specific reasons. Discrepancies will be resolved via referencing the original article and via group discussions or in consultation with the principal investigator (YL). The whole process of study selection is summarized as a flowchart in Figure 1.

#### 2.4.2 Data extraction

Two independent investigators (YYL and YJW) will extract and tabulated all data using a standardized data extraction form. Discrepancies will be resolved via referencing the original article and in consultation with the principal investigator (YL).

The following data will be extracted including leading author, year of publication, journal, country or region, study design, sample size, patients age, diagnostic criteria of PCOS, type of intervention, controls, treatment frequency, outcome measures, adverse effects, and any other relevant information.

#### 2.4.3 Assessment of risk of bias in included studies

Reviewers will use the Cochrane risk of bias tool to examine the following 7 domains: random sequence generation, allocation concealment, blinding of participants and personnel, blinding of outcome assessment, incomplete data assessment, selective outcome reporting, and other sources of bias. Each bias domain will be addressed as unclear, low, and high degrees.

### 2.5 Statistical analysis

We will use Review Manager Software (RevMan) V.5.3 for data synthesis, meta-analysis. Continuous variables will be reported as mean difference (MD) with 95% confidence intervals (CIs). For different measurement scales, standardized MD (SMD) analysis with 95% CI will be used. Categorical variables will be summarized as risk ratios (RRs) or odds ratio (OR) with 95% CIs. All analysis will be performed based on the Cochrane Handbook for Systematic Reviews of Interventions. A meta-analysis will be performed when the results of the merger are homogeneous insufficient studies.

### 2.6 Subgroup analysis

Subgroup analysis will be conducted based on the difference of interventions, controls, outcome measurements, and so on if necessary.

### 2.7 Sensitivity analysis

Sensitivity analysis will be undertaken to check the stability of merged outcome results by excluding studies with a high risk of bias if significant heterogeneity exists.

### 2.8 Quality of evidence

The quality of evidence for outcomes will be assessed with the Grading of Recommendations Assessment, Development, and Evaluation (GRADE) approach.^[16]^

The evaluation included bias risk; heterogeneity; indirectness; imprecision; publication bias. And each level of evidence will be made as very low, low, moderate, and high.

## Author contributions

**Conceptualization:** Yan Li and Yingji Wang.

**Data curation:** Yangyang Li, Xiaoying Zheng and Yingji Wang.

**Formal analysis:** Yangyang Li.

**Methodology:** Yan Li.

**Project administration:** Yan Li.

**Writing – original draft:** Yan Li and Xiaoying Zheng.

**Writing – review & editing:** Yan Li, Yingji Wang, Yangyang Li and Xiaoying Zheng.
